# Increased Isolation and Characterization of *Shigella sonnei* Obtained from Hospitalized Children in Tehran, Iran

**DOI:** 10.3329/jhpn.v26i4.1884

**Published:** 2008-12

**Authors:** Reza Ranjbar, Mohammad M. Soltan Dallal, Malihe Talebi, Mohammad R. Pourshafie

**Affiliations:** 1 Molecular Biology Research Center, Baqiyatallah University of Medical Sciences, Tehran, Iran; 2 Department of Pathobiology, School of Public Health and Institute of Public Health Research, Tehran University of Medical Science; 3 Department of Microbiology, Pasteur Institute of Iran, Tehran, Iran

**Keywords:** Antibiotic resistance, Antibiotics, Drug resistance, Microbial, Dysentery, Bacillary, Ribotyping, *Shigella sonnei*, Iran

## Abstract

*Shigella flexneri* has been the most frequent cause of shigellosis in children in Iran. To evaluate the changes in frequency of serogroups, 302 *Shigella* species were isolated in 2003 from hospitalized children, aged less than 12 years, with acute diarrhoea in Tehran, Iran. The number of collected *S. sonnei, S. flexneri, S. boydii,* and *S. dysenteriae* isolates was 178 (58.9%), 110 (37.4%), 10 (3.3%), and 4 (1.3%) respectively. Most (94%) *S. sonnei* isolates were resistant to co-trimoxazole. They were, however, relatively or completely sensitive to 15 commonly-used antibiotics. The extracted plasmids showed 12 different profiles with two closely-related patterns constituting 70% of the total isolates. Ribotyping, using *Pvu*II, *Hind*III or *Sal*I restriction enzymes, generated a single pattern for all *S. sonnei* isolates. Data suggest that *S. sonnei* has become the predominant serogroup in children in the hospitals of Tehran.

## INTRODUCTION

*Shigella* is the major cause of diarrhoeal diseases in both developing and developed countries (1). In some developing countries, it was made up of 10% of all diarrhoeal cases during the 1990s among children aged ≤5 years (2).

Of the *Shigella* species, *Shigella flexneri* and *S. sonnei* are the most prevalent serogroups found in developing and industrialized countries respectively. *S. dysenteriae* is seen mostly in South Asia and sub-Saharan Africa, and *S. boydii* has been reported worldwide with about 4% of the total shigellosis cases (1).

For many years, *S. flexneri* has been the predominant isolate in Iran (3,4). The present study was conducted to examine the prevalence of *Shigella* spp., antibiotic susceptibility patterns, and genetic characterization of *S. sonnei* isolates. We report here for the first time that *S. sonnei* was the most frequent isolate among shigellosis cases in children in Tehran.

## MATERIALS AND METHODS

### Patients

The study included all patients, aged less than 12 years, with diarrhoea (three times or more watery or soft defaecations per 24 hours that had lasted for ≤7 days, fever, abdominal pain, tenesmus with or without nausea, and vomiting), who were admitted to three large hospitals: Children Medical Center, Mofid Hospital, and Millad Hospital, in Tehran, Iran, during 2003.

A single specimen was obtained from each patient, and rectal swabs were collected from patients on the day of admission at the hospital. When the isolates were identified as *Shigella* by the conventional methods (5), these were serotyped using slide agglutination with specific antisera (MAST Group LTD, Merseyside, UK).

### Testing of antimicrobial susceptibility

Antimicrobial susceptibility test was performed according to the standard guideline of the Clinical and Laboratory Standards Institute (6) using 16 antibiotic discs (Becton Dickinson and Company, Sparks, MD, USA), such as ampicillin (10 μg), cefixime (5 μg), cefotaxime (30 μg), ceftazidime (30 μg), ceftizoxime (30 μg), cephalothin (30 μg), cephalexine (30 μg), amikacin (30 μg), gentamicin (10 μg), kanamycin (30 μg), ciprofloxacin (5 μg), nalidixic acid (30 μg), chloramphenicol (30 μg), nitrofurantoin (300 μg), furazolidone (100 μg), and co-trimoxazole (1.25/23.75 μg). *Escherichia coli* ATCC 25922 was used as a quality-control strain.

### Plasmid profiling

A high-pure plasmid isolation kit (Roche, Mannh-eim, Germany) was used for isolating bacterial plasmids as per the instructions of the manufacturer. Extracted plasmids were then separated on a 0.8% agarose gel in Tris-borate-EDTA buffer (TBE×1) (pH 8.2) by electrophoresis. The strains were grouped depending on the pattern of the plasmid DNA bands. The banding patterns were interpreted by Dice analysis and clustered by the unweighted pair group method with arithmetic averages (UPGMA) with Gelcompar II, version 4.0 (Applied Maths, Sint-Matens-latem, Belgium).

### Ribotyping

Ribotyping was performed using standard methods as reported in the previous studies (7). Bacterial DNA was digested with restriction enzymes (*Pvu*II, *Hind*III, *Sal*I) under the conditions recommended by the manufacturer (Roche Diagnostics, Mannheim, Germany). Digested DNA fragments were resolved on a 0.8% agarose gel in Tris-borate-EDTA buffer (pH 8.2) and then transferred onto nylon membrane by the alkali-blotting procedure with a vacuum blotter. Hybridization was performed with the probes labelled with digoxigenin-11-dUTP (DIG) (7). The membranes were then visualized by adding alkaline phosphate-conjugated anti-digoxigenin antibody (Roche Diagnostic GmbH, Mannheim, Germany) and 5-bromo-4-chloro-3-indolyl phosphate substrate and nitroblue tetrazolium. *Citrobacter koseri* strain CIP 105177 (collection: de l'Institut Pasteur) DNA was cleaved by *Mlu*I restriction endonuclease, and the fragments were used as molecular size standards.

## RESULTS

Of 3,050 patients with acute diarrhoea, 302 were diagnosed as having shigellosis based on clinical presentations and laboratory findings. The isolated *Shigella* strains were distributed thus: *S. sonnei* 178 (58.9%), *S. flexneri* 110 (36.4%), *S. boydii* 10 (3.3%), and *S. dysenteriae* 4 (1.3%).

Results of further examination of the *S. sonnei* strains showed that most (≥94%) *S. sonnei* isolates were resistant to co-trimoxazole, and ≤6% of the isolates were resistant to nalidixic acid, ampicillin, chloramphenicol, cefixime, and kanamycin. None of the tested isolates was resistant to ceftizoxime, ceftazidime, gentamicin, ciprofloxacin, amikacin, furazolidone, cephalothin, cefotaxime, cephalexine, and nitrofurantoin. Only 2.6% of the isolates were resistant to ≥3 antibiotics (Table).

**Table.** Plasmid profiles and antimicrobial resistance patterns of *S. sonnei*

Plasmid pattern	% of isolates	Resistance pattern (%)	Resistance phenotype
P1	ATCC 9290*	-	-
P2	1.3	R4 (1.3)	SXT, K
P3	38.7	R1 (32.5)	SXT
		R2 (5)	SXT, NA
		R4 (1.3)	SXT, K
P4	6.2	R1 (5)	SXT
		R3 (1.3)	K
P5	1.3	R1 (1.3)	SXT
P6	31.2	R1 (28.8)	SXT
		R7 (1.3)	AM, SXT, K
		R5 (1.3)	SXT, CFM
P7	3.8	R1 (3.8)	SXT
P8	3.8	R1 (3.8)	SXT
P9	1.3	R6 (1.3)	SXT, NA, C, K
P10	2.5	R1 (2.5)	SXT
P11	6.2	R1 (6.2)	SXT
P12	1.3	R1 (1.3)	SXT
P13	2.5	R1 (2.5)	SXT

*ATCC 9290 type strain was sensitive to all antibiotics; AM=Ampicillin; C=Chloramphenicol; CFM=Cefixime; K=Kanamycin; NA=Nalidixic acid; SXT=Co-trimoxazole

Plasmid analysis of clinical isolates of *S. sonnei* resulted in 12 different plasmid profiles with 2-9 DNA bands (Fig. 1). The plasmids larger than 20 kb were not analyzed because of their instability (8,9). Furthermore, the plasmid isolation kit, used in this study, is suitable for purification of small plasmids. Figure 1 shows that some DNA bands (5.1 and 2.0 kbp) were evident in most strains. No similarity in the plasmid pattern between our clinical isolates and ATCC type strain 9290 was observed. The *S. sonnei* isolates containing the plasmid profile labelled as P2 and P13 harboured the lowest (3) and the highest (9) number of DNA bands respectively. P3 (39%) was the dominant type of plasmid profile, followed by P6 (31%) (Table).

**Fig.**
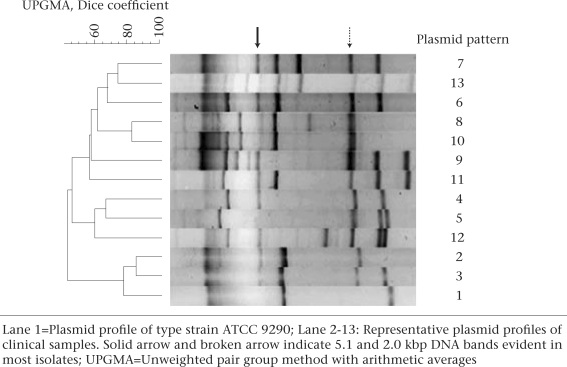
A UPGMA dendrogram showing distribution of plasmid profiles of S. *sonnei* isolates

**Fig. 2.**
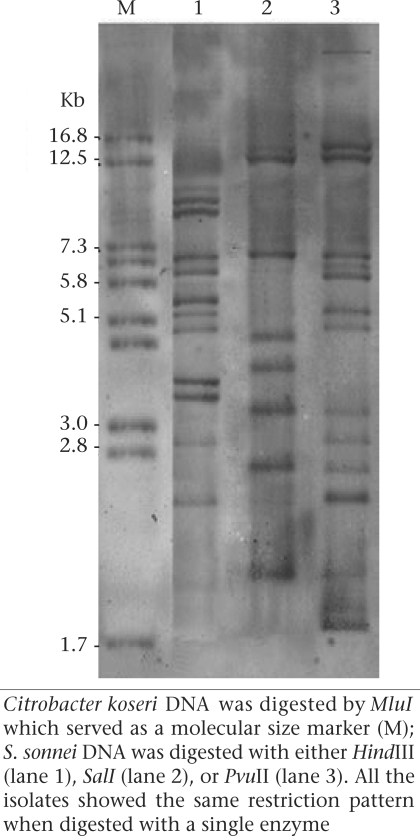
Ribotyping of S. *sonnei* strain

Ribotyping was performed using three restriction enzymes, including *Pvu*II, *Hind*III, and *Sal*I, for all the *S. sonnei* isolates (Fig. 2). Ribotyping using *Sal*I produced seven fragments ranging from 2.0 to 12.5 kbp. The highest number of DNA fragments was obtained when DNA was digested with *Pvu*II restriction enzyme, resulting in 13 fragments ranging from 1.8 to 14 kb. *Hind*III showed 11 bands each from 2.4 to 10 kbp. Only a single ribotype pattern was observed using each of the restriction enzymes.

## DISCUSSION

*S. sonnei* has been the predominant *Shigella* spp. in Europe and North America (10). Whereas, *S. flexneri* has been reported to be the most frequent *Shigella* spp. in many developing countries, including the Middle East region (11).

In the previous years, several investigators in Tehran reported that the majority (61**%**) of shigellosis cases in all age-groups were caused by *S. flexneri* (61%), followed by *S. sonnei* (31%) (3,4). The results of this study have shown that *S. sonnei* has replaced *S. flexneri* as the predominant serogroup in children aged less than 12 years in the hospitals in Tehran. More recently, Farshad and colleagues also identified *S. sonnei*as the most prevalent *Shigella* species in Shiraz, Iran, in a six-month study conducted in 2003 (12).

A multicentre study has shown that *S. flexneri* has been the most frequent isolate in Bangladesh, China, Pakistan, Indonesia, and Viet Nam (13). In the same report, *S. sonnei* has been shown as the most predominant isolate in Thailand. It was suggested that the reason for *S. sonnei* to be the principal isolate is the fact that Thailand is rapidly becoming an industrialized country (13). Similarly, Tehran is on the verge of becoming an industrialized city, and the decrease in the proportion of *S. flexneri* and an increase in *S. sonnei* may reflect the hygienic improvement in Tehran during recent years. Such a shift in the serotypes of *Shigella* has also been reported from India and Chile (14,15).

The resistance of *Shigella* isolates to the first-line antimicrobial agents has been reported in a number of countries and is increasing worldwide with increased mortality (16). With the exception of co-trimoxazole, the large majority (87%) of the isolates were relatively sensitive to 15 other antibiotics tested, indicating that the resistance of *S. sonnei* to drugs is not at an alarming rate in Iran. In the United States, the most common resistance among *S. sonnei* isolates was against ampicillin (77%) and co-trimoxazole (37%) (17). An increased resistance has been reported in developing countries, such as Chile and Bangladesh, for ampicillin (82%), co-trimoxazole (65%), and chloramphenicol (49%) (15,18).

Genetic characterization by plasmid profiling and ribotyping was also performed on our *S. sonnei* isolates. Tacket and colleagues investigated the plasmid profile of 10 *S. sonnei* isolates and found 10 different patterns (19). In other studies, 61 *S. sonnei* strains resulted in identification of 42 distinct plasmid patterns (20). These studies may suggest the existence of a large number of different plasmids in the *S. sonnei* populations. We found 12 different plasmid patterns among our *S. sonnei* isolates. The presence of a common 5.1-kbp plasmid band was evident when comparing the plasmid bands in our samples with the report by other investigators (8,21), suggesting a widespread dissemination of this plasmid DNA among *S. sonnei* isolates in different regions of the world.

Although some researchers have obtained variable results with ribotyping, the technique has been indicated to be useful for epidemiological studies of *S. sonnei* (22). Hinojosa-Ahmuda and colleagues have found six ribotypes when 100 *S. sonnei* were studied using *Sal*I restriction enzyme (23). Nastasi and colleagues have also found 13 ribotypes by examining 432 *S. sonnei* isolates (24). Several investigators have reported the presence of a single ribotype pattern in their *S. sonnei* isolates which were obtained from outbreaks or sporadic cases (9). In our study, we also observed a single ribotype pattern among the *S. sonnei* isolates collected in Tehran.

The results suggest that *S. sonnei* has replaced *S. flexneri* as the predominant serogroup in children aged less than 12 years in the hospitals in Tehran. The single ribotype dominance was also supported by the data that 70% of the isolates harboured closely-related plasmid patterns labelled as P3 and P6. The dominance of an *S. sonnei* ribotype is interesting when it is considered that the isolates were obtained from different hospitals located in a large geographical area in Tehran. The results further confirm that continuous monitoring is needed for over a prolonged period in Tehran to detect the changes in the distribution of serotypes and the antimicrobial resistance pattern of *Shigella*.

## ACKNOWLEDGEMENTS

This research was supported in part by a grant from Tehran University of Medical Sciences and Pasteur Institute of Iran.
